# Long non-coding RNAs and their potential role in predicting immunotherapy response and prognosis: a systematic review

**DOI:** 10.3389/fimmu.2026.1859747

**Published:** 2026-07-10

**Authors:** Lamia K. Alshamlani, Sara M. Alrowaydan, Nora S. Almutairi, Manar A. Alomar, Faizah M. Alotaibi

**Affiliations:** 1 College of Medicine, King Saud bin Abdulaziz University for Health Sciences, Riyadh, Saudi Arabia; 2 King Abdullah International Medical Research Center, Riyadh, Saudi Arabia; 3 College of Science and Health Professions, King Saud Bin Abdulaziz University for Health Sciences, Riyadh, Saudi Arabia; 4 Ministry of National Guard – Health Affairs, Riyadh, Saudi Arabia

**Keywords:** biomarkers, cancer, immune-checkpoint blockade, lncRNA, treatment

## Abstract

**Background:**

Immune checkpoint inhibitors (ICIs) have revolutionized cancer treatment; however, durable clinical benefit is limited to a subset of patients. Long non-coding RNAs (lncRNAs) have emerged as important regulators of tumor biology and immune responses and may serve as novel biomarkers to predict prognosis and response to ICIs.

**Methods:**

The systematic review was conducted according to PRISMA guidelines and registered in PROSPERO (CRD420251030158). Databases were searched from January 2010 to July 2025 for studies evaluating lncRNA expression in adult cancer patients treated with ICIs. Eligible studies assessed associations between lncRNAs expression and survival outcomes after ICI treatment.

**Results:**

After selection, 31 studies across multiple cancer types, were included. Most studies were retrospective bioinformatic analyses of public datasets and focused on ICI therapies. lncRNA-based signatures consistently demonstrated prognostic value by stratifying patients into groups with significantly different survival outcomes and were associated with variations in ICI response across multiple cancer types.

**Conclusions:**

LncRNAs represent promising prognostic and predictive biomarkers for ICI. Prospective validation and standardized analytical approaches are needed to support their clinical translation in precision immuno-oncology.

**Systematic review registration:**

https://www.crd.york.ac.uk/PROSPERO/, identifier CRD420251030158.

## 1 Introduction

Immune checkpoint inhibitors (ICIs) have transformed cancer treatment by restoring antitumor immune responses through targeting inhibitory immune checkpoint signaling pathways, particularly programmed cell death protein 1 (PD-1), programmed death ligand 1 (PD-L1), and cytotoxic T-lymphocyte–associated protein 4 (CTLA-4) (1). Over the past decade, their use has led to clinical benefit across several malignancies, including melanoma (2), non-small cell lung cancer (NSCLC) (3), renal cell carcinoma (4), and urothelial carcinoma (5), leading to their widespread regulatory approval (6). Despite the encouraging results, only a subset of patients can achieve durable responses, while many show primary resistance or develop acquired resistance during treatment (7). In addition, treatment can be complicated due to immune-related adverse events (irAEs) (8). Together, these limitations highlight the need for reliable biomarkers that can predict prognosis and response to ICIs, allowing for improved patient selection and treatment optimization. Currently available biomarkers, such as PD-L1 expression, tumor mutational burden, and microsatellite instability have limited accuracy and often fail to fully reflect the complexity of tumor–immune interactions (9).

**Figure 1:**
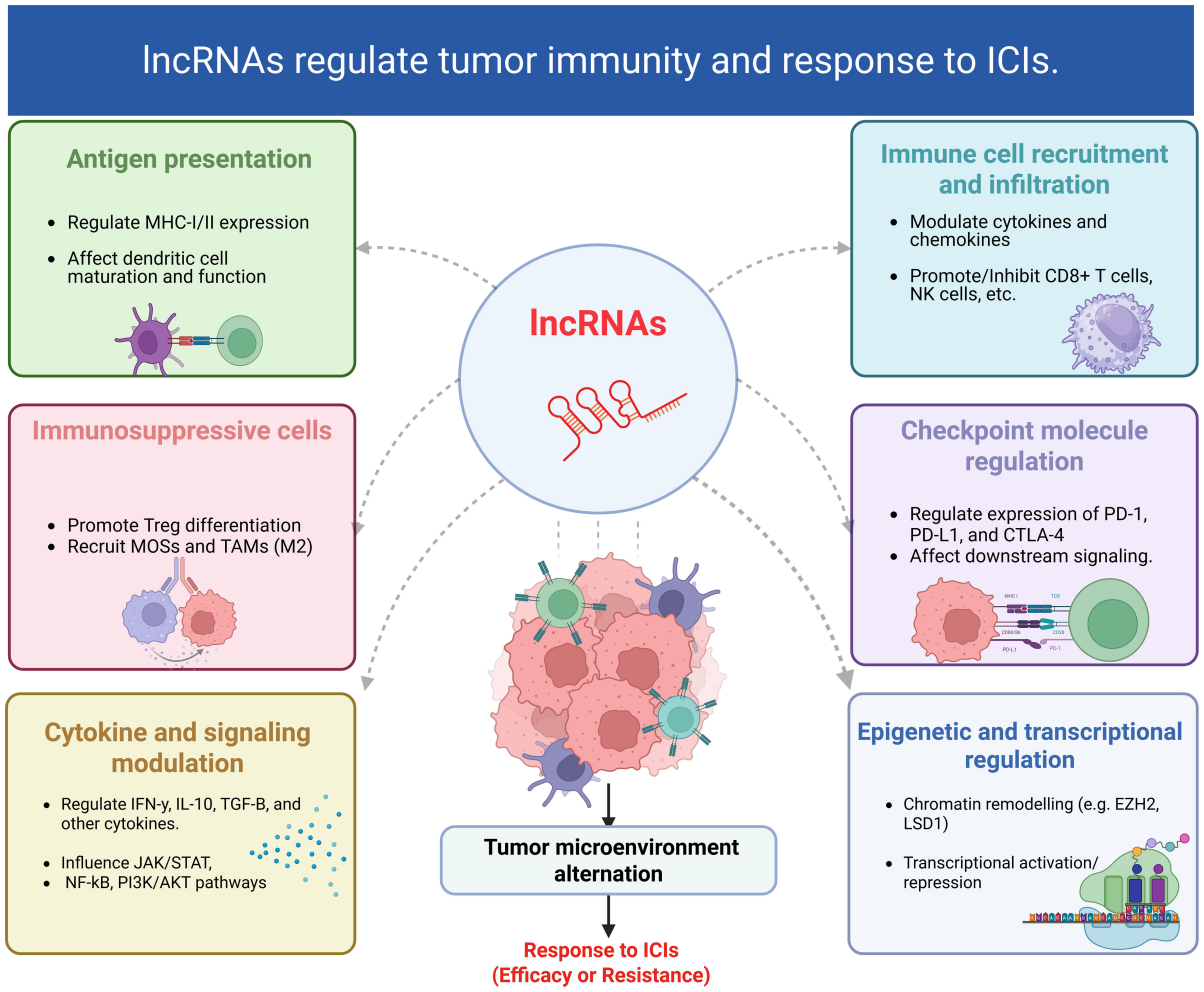
lncRNAs regulate tumor immunity and influence response to ICIs. lncRNAs are important regulators of the tumor immune microenvironment and can influence the efficacy or resistance of ICIs through multiple mechanisms. LncRNAs modulate antigen presentation by affecting MHC expression and dendritic cell function, regulate immune-cell recruitment and infiltration through cytokine and chemokine signaling, promote immunosuppressive cell populations including Tregs, MDSCs, and M2 TAMs, and influence inflammatory pathways such as JAK/STAT, NF-κB, and PI3K/AKT signaling. In addition, lncRNAs can regulate immune checkpoint molecules including PD-1, PD-L1, and CTLA-4, as well as epigenetic and transcriptional programs involved in antitumor immunity. Collectively, these mechanisms shape the tumor immune microenvironment and contribute to either enhanced sensitivity or resistance to ICI therapy. lncRNA, long non-coding RNA; ICI, immune checkpoint inhibitor; PD-1, programmed cell death protein 1; PD-L1, programmed death-ligand 1; CTLA-4, cytotoxic T-lymphocyte-associated protein 4; MHC, major histocompatibility complex; Treg, regulatory T cell; MDSC, myeloid-derived suppressor cell; TAM, tumor-associated macrophage; JAK, Janus kinase; STAT, signal transducer and activator of transcription; NF-κB, nuclear factor kappa B; PI3K, phosphoinositide 3-kinase; AKT, protein kinase B.

Long non-coding RNAs (lncRNAs) have emerged as important regulators in cancer biology (10). They are transcripts longer than 200 nucleotides that lack protein-coding capacity but can act at the epigenetic, transcriptional, and post-transcriptional levels (11, 12). In addition to their role in tumor initiation, metastasis, and therapeutic resistance, lncRNAs highlight their potential use as diagnostic and prognostic biomarkers (13, 14). Beyond their role in tumor initiation and progression, lncRNAs are increasingly recognized as key regulators of antitumor immunity. They can influence antigen presentation, immune-cell recruitment, cytokine signaling, immune checkpoint expression, and epigenetic regulation of immune-related genes, thereby shaping the tumor immune microenvironment and influencing sensitivity or resistance to immune checkpoint inhibitors (10, 15–17). The major mechanisms through which lncRNAs regulate tumor immunity and response to ICIs are summarized in **Figure 1**. More recently, growing evidence suggests a correlation between lncRNAs expression and response to ICIs. For instance, dysregulation of lncRNAs such as NEAT1 (18), HCP5 (19), and MIAT (20) have been linked to immune checkpoint regulation and response to ICI treatment. Furthermore, LIMIT (lncRNA inducing MHC-I and immunogenicity of tumor), an immunogenic lncRNA can promote tumor antigen presentation through activating the GBP–HSF1 axis (21). LncRNAs can also influence the PD-1/PD-L1 signaling through multiple mechanisms. For example, evidence suggests that SNHG12 acts as a scaffold contributing to PD-L1 mRNA stability, while SChLAP1 appears to extend PD-L1 half-life through inhibition of AUF1 interaction with the 3′UTR (22). More recent studies have continued to expand the range of immune-related lncRNA signatures. One study identified a signature involving the IDO1-MIR155HG pair that effectively predicts three- and five-year prognosis and ICI response in melanoma (23). In patients with gastric cancer, migrasome-related and disulfidptosis-related lncRNA signature have been shown to predict overall survival (24, 25). These reports highlight the potential role of lncRNAs as biomarker for ICI treatment (23, 26). However, the clinical use of lncRNAs as ICI biomarkers remains heterogeneous due to their dual roles in the cancer–immunity cycle, tissue-specific expression across malignancies, and significant variability in methodological approaches and clinical outcome reporting across studies (27). Therefore, the translational potential of lncRNAs as biomarkers for ICI treatment have not been clearly established. In this systematic review, we examined the existing literature on lncRNAs as potential biomarkers of prognosis and response to ICIs. By bringing together findings from clinical studies across different cancer types, we provide an overview of current landscape of lncRNA studies as biomarkers, summarize the main candidates that have been reported, and highlight key gaps and future directions for biomarker development in ICI-treated patients.

## 2 Methods

### 2.1 Registration

The study was conducted in accordance with the Preferred Reporting Items for Systematic Reviews and Meta-Analyses (PRISMA) guidelines (28) and registered with the International Prospective Register of Systematic Reviews (PROSPERO) and can be accessed at https://www.crd.york.ac.uk/PROSPERO/view/CRD420251030158.

### 2.2 Search strategy and study selection

A comprehensive search of literature was conducted using PubMed, Web of Science and MEDLINE from January 2010 up to July 2025. The search strategy combined Medical Subject Headings (MeSH) and free text terms related to long non-coding RNAs, immune checkpoint inhibitors, cancer, and biomarker. Search terms included, but were not limited to, “long non-coding RNA”, “lncRNA”, “immune checkpoint inhibitor”, “immune checkpoint blockade”, “PD-1”, “PD-L1”, “CTLA-4”, “cancer”, “tumor”, “malignancy”, “biomarker”, “prognosis”, “prediction”, and related synonyms. Boolean operators and database-specific indexing terms were applied where appropriate. The complete database-specific search strategies are provided in **Supplementary Table 1**. All retrieved records were imported into Rayyan software (Qatar Computing Research Institute, Doha, Qatar). After removal of duplicates, two investigators (NSA and SMA) independently conducted the initial screening which included title and abstracts. Full-text articles were then independently assessed for eligibility by two investigators (LKA and MAA). Disagreements were resolved by discussion with a senior investigator (FMA).

### 2.3 Eligibility criteria

The eligibility criteria were predefined in the study protocol and structured according to the PICOS framework (Population, Intervention, Comparator, Outcomes, and Study design). The following inclusion criteria were applied: 1- Population: Adult patients with histologically or molecularly confirmed solid or hematological malignancies at any stage. 2- Intervention: Treatment with ICIs, either as monotherapy or in combination with other systemic therapies. 3- Comparator: Clinically relevant comparison groups defined within individual studies, including high versus low lncRNA expression, high-risk versus low-risk lncRNA signature groups, responders versus non-responders to immune checkpoint inhibitor therapy. 4- Outcomes: Evaluation of lncRNA biomarkers in relation to clinical outcomes, including OS, progression-free survival (PFS), or treatment response ICI as the primary reported endpoints. 5- Study design: Eligible studies included full-text, English-language. Studies that were narrative reviews, editorials, case reports, conference abstracts, *in vitro* studies, animal studies, or studies involving non-malignant conditions or lacking clear clinical outcomes were excluded from the review. In addition, studies that did not specifically assess the association between lncRNA expression and prognosis or response to ICIs were excluded.

### 2.4 Data extraction

Data from included studies were extracted and imported into Microsoft Excel spreadsheet (Microsoft, Redmond, WA, United States). The following variables were extracted when available: title, first author, publication date, study duration, study design, study databases, location of the study, total sample size, age, gender, tumor type, cancer stage, type of ICI used, long non-coding RNAs, lncRNAs expression direction, biological sample Source, comparator type, description of comparator treatment or group, comparator sample type, clinical outcomes (PFS and OS), association of lncRNAs with treatment response or disease progression (predictive and prognostic). All variables were extracted by four investigators (MAA, LKA, NSA, and SMA) and then reviewed by all investigators (MAA, LKA, NSA, SMA and FMA).

### 2.5 Risk of bias assessment

The methodological quality and risk of bias of the included studies were assessed using the Newcastle-Ottawa scale (NOS) (29). This scale assesses the quality across three categories including study group selection, comparability, and ascertainment of clinical outcomes, with a maximum of eight points. Four investigators assessed the quality of each study independently (MAA, LKA, NSA, and SMA), with a fifth senior investigator (FMA) assessing any discrepancies. The scale was used to assess quality rather than as a form of exclusion. Any study scoring seven points or higher was considered to be of high quality (**Supplementary Figure 1**).

## 3 Results

### 3.1 Description of included studies

**Figure 2:**
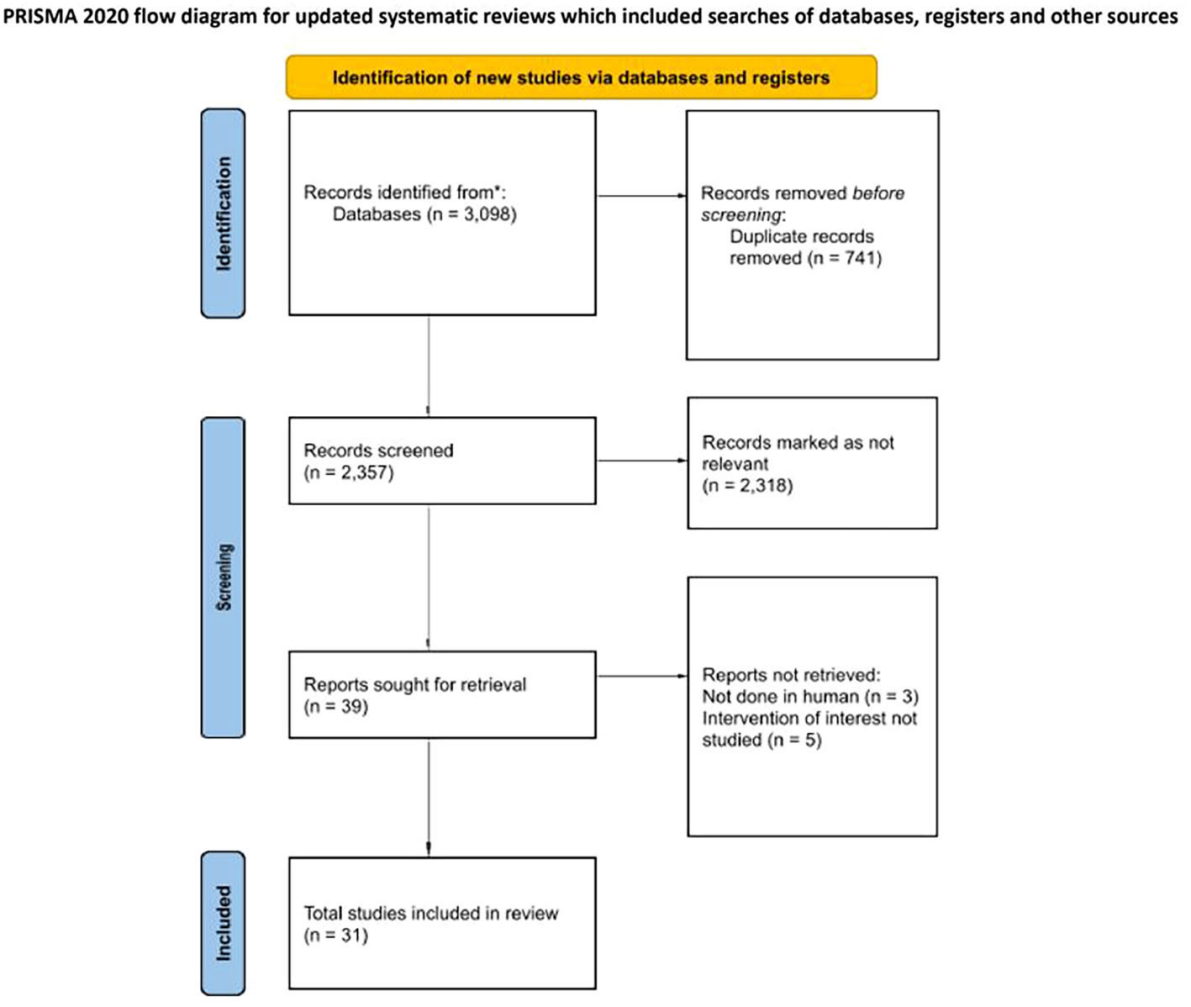
PRISMA flow diagram of study selection process. Flow diagram illustrating the study selection process according to the Preferred Reporting Items for Systematic Reviews and Meta-Analyses (PRISMA) guidelines. A total of 3,098 records were identified through database searching. Following removal of duplicate records and screening of titles and abstracts, potentially eligible articles underwent full-text assessment. Studies that met the predefined inclusion criteria were included in the qualitative synthesis. Reasons for exclusion at the full-text review stage are presented within the diagram.

The study selection process is summarized in the PRISMA flow diagram (**Figure 2**). A total of 3,098 studies were initially retrieved from the databases. Following title and abstract screening, full-text assessment and removal of duplicates, 31 studies published between 2020 and 2025 met the predefined inclusion criteria and were included in the final analysis. The baseline characteristics of included studies are presented in (**Table 1**). Out of a total of 31 included studies, 28 were retrospective study design. The 31 included studies were conducted in different countries including the majority in China (n = 24), followed by the United States (n = 1), Greece (n = 1), and Spain (n = 1) (**Table 1**). The remaining studies were conducted multinational (n = 4). Nearly all studies (28 of 31) relied on bioinformatic analyses of publicly available datasets, most commonly The Cancer Genome Atlas (TCGA), Gene Expression Omnibus (GEO), and other publicly accessible genomic repositories in addition to external independent cohorts (**Table 2**).

**Table 1:** Baseline characteristics of studies included.

Study ID	Country of origin	Study design	Age (median ± SD)	Gender	Cancer type	Stage	ICI type
Studies that utilized clinical cohorts							
Wang et al., 2023 (30)	China	Prospective	64 (51-71)	Male: 6 (85.71)	NSCLC	IV, IIIB	Anti-PD-1
Oliver et al., 2022 (31)	Spain	Prospective	M: 62.74	Male: 6 (50%)	Metastatic melanoma	I, II, III, IV	Anti-PD-1
Katifelis et al., 2025 (32)	Greece	Prospective	M: 65.6 ± 11	Male: 25 (81%)	mccRCC	Metastatic	Anti-PD-1 and anti-CTLA-4
Studies that utilized computational Validation Cohorts Only							
Shen et al., 2022 (33)	China/Germany	Retrospective	>60 (52.7%)	NR	LUAD	Early and advanced stages	Anti-PD-L1 and Anti–PD-1
Zhu et al., 2023 (34)	China	Retrospective	Validation cohort 1: 61 (51-69) Validation cohort 2: 62 (52-69)	Male: 245 (67.31%)	HCC	I, II, III, IV	ICI-treated cohort (agent not reported)
Liu et al., 2022 (35)	China	Retrospective	TCGA mean: 68.1 years GEO mean: 65.1 years	TCGA: Male: 303 GEO: Male: 135	Bladder	I, II, III, IV	Anti-PD-1, Anti-PD-L1, Anti-CTLA-4
Zhou et al., 2021 (36)	China/Germany	Retrospective	<60: 15 (37%)	Male: 26 (63%),	Advanced melanoma	Advanced stage	Anti-PD-1, Combined anti-PD-1 and anti-CTLA-4
Wang et al., 2024 (37)	China	Retrospective	>60: 35	Male: 81	ESCC	I, II, III, IV	Anti-PD-1
Yu et al., 2020 (38)	China	Retrospective	Bladder: 62; melanoma: 58.3	Bladder (TCGA): Female: 106 (26.1%) Melanoma (TCGA): Female: 37 (52.1%)	Bladder + melanoma + multicancer	I, II, III, IV	Anti-PD-L1, Anti-PD-1 Anti-CTLA-4,
Yin et al., 2024 (39)	China/Germany	Retrospective	M: 60.91	Male: 385 (73.06%)	HNSCC	I, II, III, IV	Anti-PD-1, and anti-PD-L1
Ma et al., 2022 (40)	China	Retrospective	NR	Male:361	NSCLC + melanoma	Advanced/metastatic (derived from source cohorts)	Anti-PD-1
Cao et al., 2022 (41)	China	Retrospective	< 60: 218 (44.5%)	Male: 361	HNSCC	I, II, III, IV	Anti-PD-1
Zhong et al., 2024 (42)	China	Retrospective	NR	NR	LUAD	I, II, III, IV	Anti-PD-L1
Xiao et al., 2022 (43)	China	Retrospective	Old: 295 Young: 108	Male: 298	Bladder	I, II, III, IV	PD-L1 inhibitor
Hui et al., 2023 (44)	China	Retrospective	NR	NR	Bladder	I, II, III, IV (derived from source cohorts)	Anti-PD-L1, Anti-CTLA-4
Qu et al., 2024 (45)	China	Retrospective	NR	NR	Multi-cancer	I, II, III, IV (derived from source cohorts)	Anti-PD-1 Anti-PD-L1 Anti-CTLA-4
Hu et al., 2021 (46)	China	Retrospective	>65:106	Male: 151	Adenocarcinoma of the esophagogastric junction	I, II, III, IV	Anti-PD-L1
Zhou et al., 2024 (47)	China	Retrospective	NR	Female: 100%	Ovarian	I, II, III, IV (derived from source cohorts)	Anti-PD-1, anti-PD-L1 and anti-CTLA-4
Xiao et al., 2021 (48)	China	Retrospective	NR	NR	Gastric	I, II, III, IV (derived from source cohorts)	Anti-PD-L1
Li et al., 2023 (49)	China	Retrospective	Mean ~68 yrs	High risk: Male: 147 (36.9 %). low risk: Male:146 (36.7 %)	Bladder	I, II, III, IV	Anti-PD-1, anti-PD-L1 and anti-CTLA-4
Wu et al., 2020 (50)	China	Retrospective	≤ 70 years: 229 patients >70 years: 176 patients	Male: 300 (74%)	Bladder	I, II, III, IV	Anti-PD-1
Studies that utilized computational validation cohort in addition to clinical Cohort							
Ma et al., 2021 (51)	China/USA	Retrospective	>60 (52.7%)	TCGA test: Male: 151 (75.5%), FUSCC: Male:70 (87.5%), GSE41613: Male: 66 (68%) and GSE4274: Male:58 (78.4%)	HNSCC	I, II, III, IV	Anti-PD-1
An et al., 2025 (52)	China	Retrospective	< 60: 7	Male: 13	Gastric cancer	I, II, III	Anti-PD-1
Xue et al., 2022 (53)	China	Retrospective	TCGA:>65: 256 Clinical cohort 1:>65: 6 (35.3%) Clinical cohort 2: Mean: 53.1 years	Male: 237(46.2%)	LUAD	I, II, III, IV (derived from source cohorts)	NR
Toker et al., 2023 (18)	USA	Retrospective	Melanoma range: 19.0-84.0. Glioblastoma range: 30.31-72.86	Melanoma, Male: 19. Glioblastoma, Male: 9	Melanoma; GBM	Melanoma: Metastatic, GBM: Recurrent	Anti-PD-1
Ren et al., 2024 (54)	China	Retrospective	NR	NR	HNSCC	III, IV	Anti-PD-1, anti-PD-L1 and anti-CTLA-4
Zhang et al., 2021 (55)	China	Retrospective	68.0 ( ± 10.6)	TCGA: Male: 297 (73.9%)	Bladder Cancer	I, II, III, IV	PD-L1 inhibitor
Song et al., 2022 (56)	China	Retrospective	M: 48.7 (29-64)	Male: 678	Colorectal	I, II, III, IV	Anti-PD-L1
Yang et al., 2024 (57)	China	Retrospective	>65: 250	Male: 304	Bladder	I, II, III, IV	Anti-PD-1, anti-PD-L1 and anti-CTLA-4
Jiang et al., 2021 (58)	China	Retrospective	>65: 205	Male: 238	Gastric	I, II, III, IV	Anti-PD-L1
Zhang et al., 2024 (59)	China	Retrospective	GSE19234: 63.3 years GSE19293: NR GSE22153: 56.2 years GSE65904: 62.3 years GSE115978: 67 years Clinical cohort: NR	GSE19234: Male: 28 GSE19293: Male: 25 GSE22153: Male: 31 GSE65904: Male: 124 GSE115978: Male: 9 Clinical cohort: Male: 13	Melanoma	III, IV	Anti-PD-L1

NSCLC, Non-Small Cell Lung Cancer; Anti-PD-1, Anti-Programmed Cell Death Protein 1; GSE, Gene Expression Omnibus Series; LUAD, Lung Adenocarcinoma; Anti-PD-L1, Anti-Programmed Death-Ligand 1; HCC, Hepatocellular Carcinoma; HNSCC, Head and Neck Squamous Cell Carcinoma; Anti-CTLA-4, Anti-Cytotoxic T-Lymphocyte-Associated Protein 4; ESCC, Esophageal Squamous Cell Carcinoma; GBM, Glioblastoma; mccRCC, Metastatic Clear Cell Renal Cell Carcinoma; NR, not reported in the original publication despite review of the full text and **Supplementary Materials**.

**Table 2:** Development and external validation cohorts of included lncRNA biomarker studies.

Study	Development dataset and sample size	Validation dataset	Study objective
Studies that utilized clinical cohort			
Wang et al., 2023 (30)	Clinical cohort: 7	None	Both prognostic and predictive
Oliver et al., 2022 (31)	Clinical cohort: 16	None	Both prognostic and predictive
Katifelis et al., 2025 (32)	Clinical cohort: 31	None	Both prognostic and predictive
Studies that utilized computational validation cohort only			
Shen et al., 2022 (33)	TCGA: 585, GSE4345: 110	IMvigor210 cohort: NR, GSE78220: NR	Both prognostic and predictive
Zhu et al., 2023 (34)	TCGA + GEO: 364	Randomly two split validations	Both prognostic and predictive
Liu et al., 2022 (35)	TCGA: 430	GSE13507: 256	Both prognostic and predictive
Zhou et al., 2021 (36)	PRJNA356761: 51	PRJEB23709: 41	Both prognostic and predictive
Wang et al., 2024 (37)	TCGA: 81	GEO: 120	Both prognostic and predictive
Yu et al., 2020 (38)	IMvigor210 cohort: 348	Melanoma (TCGA): 71. bladder (TCGA): 406, additional TCGA pancancer multicohort: 2951	Predictive only
Yin et al., 2024 (39)	TCGA: 527	GEO cohorts (META cohort): 551, GSE78220: 28, GSE91061: 105 GSE135222: 27, IMvigor210 cohort: 348	Both prognostic and predictive
Ma et al., 2022 (40)	GSE135222, GSE126044: NR	GSE78220, GSE131907, PRJEB23709: NR	Both prognostic and predictive
Cao et al., 2022 (41)	TCGA: 490	Randomly two split validations: NR	Both prognostic and predictive
Zhong et al., 2024 (42)	TCGA: 494	Randomly two split validation, IMvigor210 cohort: NR	Prognostic only
Xiao et al., 2022 (43)	TCGA: 403	IMvigor210 cohort: 48	Both prognostic and predictive
Hui et al., 2023 (44)	IMvigor210, TCGA: NR	GSE31684: NR	Both prognostic and predictive
Qu et al., 2024 (45)	TCGA: 8401, GTEx: 323	Multiple ICI treated cohort: 161	Both prognostic and predictive
Hu et al., 2021 (46)	TCGA + UCSC Xena: 201	TCGA randomly two split validations: 61, IMvigor210 cohort: 348	Prognostic only
Zhou et al., 2024 (47)	TCGA: 379 tumors + 88 normal tissues	GSE9891: 273	Both prognostic and predictive
Xiao et al., 2021 (48)	TCGA: 300	GSE62254: 300, GSE84437: 433, GSE14549: 192	Both prognostic and predictive
Li et al., 2023 (49)	TCGA: 398	IMvigor210 cohort	Both prognostic and predictive
Wu et al., 2020 (50)	TCGA: 405	Randomly two split validations	Both prognostic and predictive
Studies that utilized computational validation cohort in addition to clinical Cohort			
Ma et al., 2021 (51)	TCGA: 498	GSE41613: 97, GSE42743: 74, clinical cohort: 80	Both prognostic and predictive
An et al., 2025 (52)	TCGA: 340	TCGA randomly two split validations: 170, PRJEB25780 immunotherapy cohort: 45, clinical cohort: 23	Both prognostic and predictive
Xue et al., 2022 (53)	TCGA: 598	Clinical cohort 1: 17, clinical cohort 2: 6	Both prognostic and predictive
Toker et al., 2023 (18)	Melanoma pilot RNA-seq dataset (ICB-treated patients; pilot cohort)	GBM GSE121810 (n=24 recurrent GBM patients treated with pembrolizumab; IDH-WT adjuvant subgroup n=13); Dana-Farber Cancer Institute GBM cohort (n=28 IDH-WT GBM patients on PD-1/PD-L1 ICB; n=18 after FFPE exclusion)	Both prognostic and predictive
Ren et al., 2024 (54)	TCGA: 545	GSE65858: 270, clinical cohort: 72	Both prognostic and predictive
Zhang et al., 2021 (55)	TCGA: 402	IMvigor210: 348, clinical cohort: 10	Both prognostic and predictive
Song et al., 2022 (56)	GSE39582: 579, GSE17536: 177, GSE38832: 122	TCGA: 486, clinical cohort: 6	Both prognostic and predictive
Yang et al., 2024 (57)	TCGA: 204	TCGA randomly two split validations: 202, GSE39281: 94, Imvigor21: 348, clinical cohort: 65	Both prognostic and predictive
Jiang et al., 2021 (58)	TCGA: 187	TCGA randomly two split validations: 184, clinical cohort: 29, Imvigor210: 348	Both prognostic and predictive
Zhang et al., 2024 (59)	TCGA: 461 tumors + 558 normal tissues	GSE22153, GSE22154, GSE19234, GSE19293, GSE65904, GSE99898 Hospital cohort: 20 melanoma, 5 normal, Clinical samples: 24 tumour and 8 normal samples	Both prognostic and predictive

TCGA, The Cancer Genome Atlas; GEO, Gene Expression Omnibus; GSE, Gene Expression Omnibus Series; GTEx, Genotype Tissue Expression; GBM, Glioblastoma; Anti-PD-1, Anti-Programmed Cell Death Protein 1; Anti-PD-L1, Anti-Programmed Death-Ligand 1; UCSC Xena, University of California, Santa Cruz Xena platform; PRJEB25780, PRJNA356761 and PRJEB23709, Public BioProject transcriptomic datasets; NR, not reported in the original publication despite review of the full text and **Supplementary Materials**.

### 3.2 Patient characteristics

**Figure 3:**
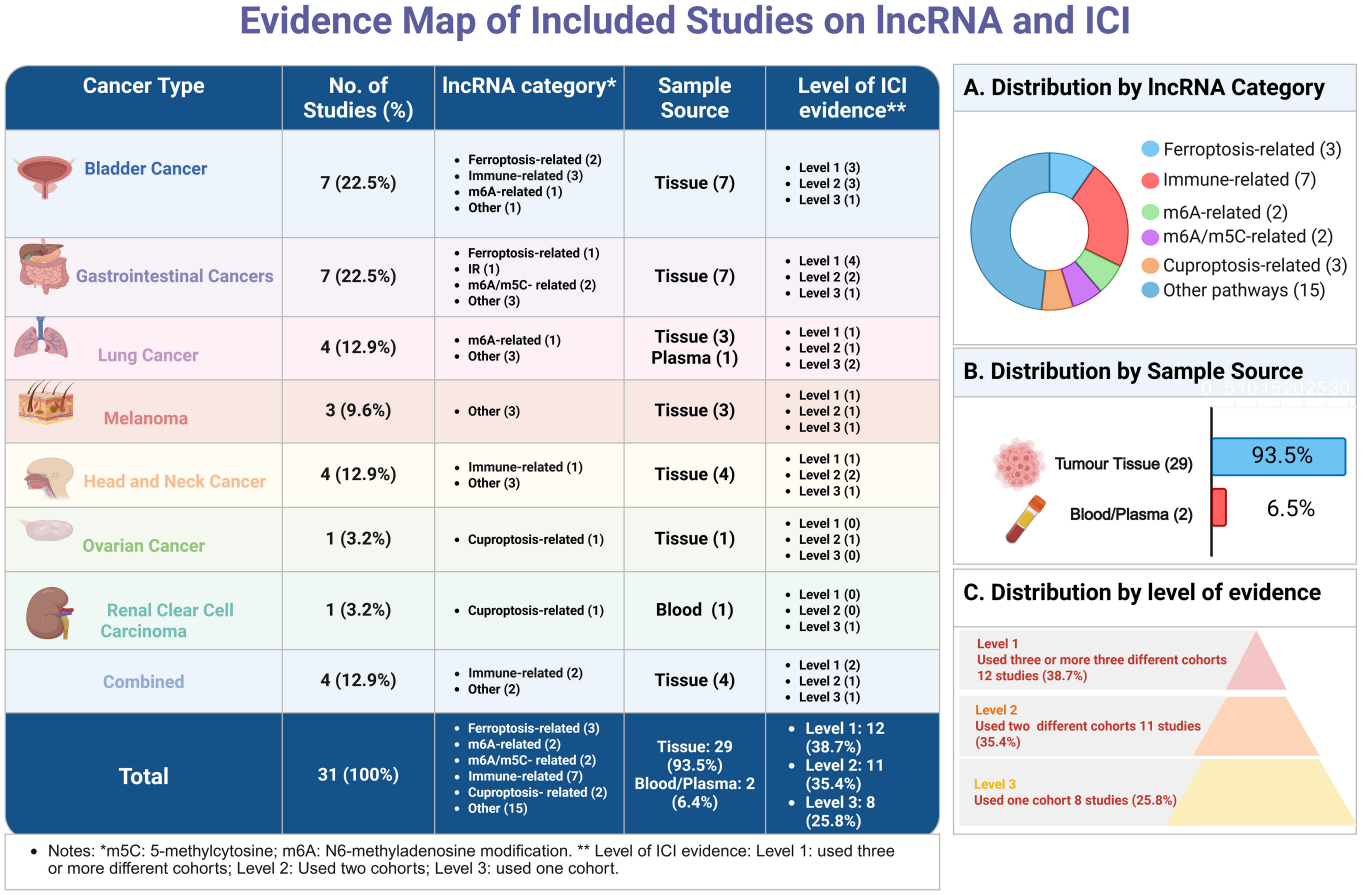
Evidence Map of Studies Evaluating lncRNAs as Potential Biomarkers in Patients Treated with ICIs. The evidence map summarizes the characteristics of the 31 studies included in this systematic review. The left panel presents the distribution of studies according to cancer type, number of studies, lncRNA category, biological sample source, and level of ICI-specific evidence. The right panels summarize the overall distribution of lncRNA categories **(A)**, biological sample sources **(B)**, and levels of evidence **(C)**. Studies were categorized according to the strength of ICI-specific evidence: Level 1, studies incorporating three or more independent immunotherapy cohorts; Level 2, studies utilizing two independent cohorts; and Level 3, studies relying on a single cohort.

Sample sizes varied across the included studies. The majority of patients were older adults (≥60 years), male patients, and individuals with advanced-stage disease, most commonly stages III and IV (**Table 1**). In most studies, cohorts treated with ICIs were primarily used for validation of lncRNA biomarkers. Regarding treatment regimens, anti–PD-1 and anti–PD-L1 therapies were the most frequently evaluated ICIs. A subset of studies also investigated anti–CTLA-4 therapies, as well as combination treatment regimens (**Table 1**). To provide an overview of the available evidence, an evidence map was constructed summarizing the distribution of included studies according to cancer type, lncRNA category, sample source, and level of ICI-specific evidence (**Figure 3**). Bladder cancer (35, 43, 44, 49, 50, 55, 57) and gastrointestinal cancers (34, 37, 46, 48, 52, 56, 58) represented the most frequently investigated malignancies, followed by four on lung cancer (30, 33, 42, 53), four on head and neck cancer (39, 41, 51, 54), three on melanoma (31, 36, 59). Additional studies investigated multiple cancer types (18, 38, 40, 45), while the remaining studies examined ovarian cancer (47) or renal clear cell carcinoma (32). Tumor tissue was the predominant biological sample source. Immune-related lncRNA signatures constituted the most commonly evaluated biomarker category, although studies also investigated ferroptosis-, m6A/m5C-, and cuproptosis-related lncRNA signatures. The level of ICI-specific evidence varied considerably across studies, highlighting the heterogeneity of the current literature (**Table 1**) and (**Figure 3**).

### 3.3 lncRNA biomarker models and analytical approaches

The characteristics of lncRNA biomarkers and analytical approaches are detailed in (**Table 3**). A dominant methodological pattern was the development of multi-lncRNA signatures, typically constructed using penalized regression models (e.g., LASSO) and Cox proportional hwazards analysis. These signatures created risk scores that classified patients into high-risk and low-risk groups and only a few studies reported on individual lncRNA (18, 59, 60). The majority of studies used tumor tissue transcriptomes, while fewer used plasma, peripheral blood, or extracellular vesicle–derived RNA.

**Table 3:** Summary of lncRNA signature, mechanisms, and associations with ICI response.

Study ID	Key pairs lncRNA/signature	Mechanism (expression & ICI association)	Sample source	Method used
Clinical cohort				
Wang et al., 2023 (30)	lnc-CENPH-1, lnc-CENPH-2, lnc-ZFP3-3	High expression → poor ICI response	Plasma exosomal RNA	Pearson’s Correlation Coefficient.
Oliver et al., 2022 (31)	ceRNA network–associated lncRNAs incl. 69 lncRNAs	High risk → poor OS/PFS	Tumor biopsies	Cox
Katifelis et al., 2025 (32)	Cuproptosis-related lncRNAs: FOXD2-AS1 MINCR LINC02154	Upregulation during ICI treatment → progressive disease & poor immunotherapy response	Peripheral blood	qRT-PCR
Studies that utilized computational validation cohort only				
Shen et al., 2022 (33)	TMPO.AS1, OGFRP1, LINC01117, HIF1A.AS1, LINC00592, WWC2.AS2, TARID, LINC00628 and ABCA9.AS1	High score → better OS & predicted ICI benefit	Tumor transcriptomes	WGCNA, and Cox
Zhu et al., 2023 (34)	CD4+ Tconv-related lncRNA risk signature: AC012073.1, AL031985.3, LINC01060, MKLN1-AS, MSC-AS1 and TMCC1-AS1	High score → poor OS & predicted worse ICI response	Tumor tissue	LASSO & Cox
Liu et al., 2022 (35)	Ferroptosis-related lncRNA risk signature	High score → poor OS & poorer immunotherapy response	Tumor tissue	Univariate and multivariate Cox regression, GSEA
Zhou et al., 2021 (36)	AC010904.2, LINC01126, AC012360.1, AC024933.1, AL442128.2, AC022211.4, AC022211.2, AC127496.5, NARF-AS1, AP000919.3, AP005329.2, AC023983.1, AC023983.2, AC139100.1 and AC012615.4	High score → lower response & poor survival on anti-PD-1	Tumor biopsies	WGCNA + LASSO
Wang et al., 2024 (37)	m6A/m5C-related lncRNA RiskScore: LINC00847, LINC00942, TTTY15, LINC01602, LINC01310, LINC00898, EGFR-AS1, MIF-AS1, LINC00993 and LINC01415	Low RiskScore → better OS & predicted ICI benefit	Tumor transcriptomes	LASSO + Cox
Yu et al., 2020 (38)	29-lncRNA weighted immune-related lncRNA score signature (core driving lncRNAs include NKILA, AC002116.2, AP000251.1, and TMEM147-AS1)	Low score → longer OS & better ICI response	Tumor RNA-seq	Clustering + multiomic
Yin et al., 2024 (39)	CMPIS (16 genes incl. lncRNAs)	Low CMPIS → “hot” tumors & predicted ICI sensitivity	Multi-omics tumor data	Cox
Ma et al., 2022 (40)	AE000661.37, XLOC_020141, XLOC_033882 and LOC105369334.	High risk → poorer OS/PFS & lower ICI response	Tumor RNA-seq	LASSO + Cox
Cao et al., 2022 (41)	31-lncRNA immune-related classifier	Defines two HNSCC subtypes: C2 “immune-hot” subtype → higher predicted PD-1 sensitivity; C1 (immune-cold) shows lower immune activity.	Tumor transcriptomes	NMF + LASSO
Zhong et al., 2024 (42)	PAM-SRFLncSig (PI3K/Akt/mTOR-related lncRNA signature): High expression (AC084757.3, LINC02802, AC024896.1, LINC00941, LINC01312) and low expression (AC010999.2 and AC026979.2)	High PAM-SRFLncSig risk score → reflects activation of PI3K/Akt/mTOR-linked to poor OS, and reduced predicted benefit from ICI.	Tumor transcriptomes	LASSO + Cox
Xiao et al., 2022 (43)	AL683807.1, LINC02446, PSMB8-AS1, U62317.4 and USP30-AS1	High risk → worse prognosis	Tumor RNA-seq	LASSO + Cox + WGCNA+
Hui et al., 2023 (44)	Three-lncRNA ICI signature (TFAP2A-AS1, SBF2-AS1 and RRN3P2)	High risk → poor OS & lower ICI response; Low risk → better anti-PD-L1 response	Tumor RNA-seq	LASSO + Cox
Qu et al., 2024 (45)	Immune checkpoint–related lncRNA core regulatory circuitry (ICP-LncCRCT) network	Specific lncRNA triplets regulate immune checkpoints; favorable circuitry → better OS & ICI response	Tumor + scRNA	LASSO
Hu et al., 2021 (46)	Six-lncRNA immune signature (LINC01502, FLJ38122, C15orf32, LINC00706, LINC01348, and BCAR4)	High risk → poor OS; Low risk → predicted better ICI benefit	Tumor RNA-seq	ssGSEA + Cox
Zhou et al., 2024 (47)	Nine-lncRNA cuproptosis signature (RGMB-AS1, TYMSOS, DANCR, LINC00702, LINC00240, LINC00996, DNM1P35, LINC00892, and TMEM254-AS1)	High risk → immune-cold & poor OS; Low risk → predicted ICI benefit	Tumor RNA-seq	LASSO + Cox
Xiao et al., 2021 (48)	17-ferroptosis-related-lncRNA signature	High risk → worse prognosis	Tumor RNA-seq	LASSO + Cox
Li et al., 2023 (49)	Seven-lncRNA m7G signature (GATA3-AS1, LINC00930, LINC01341, MED14OS, MIR100HG, RUSC1-AS1, SNHG4)	High risk → ↑ PD-1/PD-L1/CTLA4 expression & predicted better anti-PD-1/PD-L1 response; however associated with worse OS	Tumor RNA-seq	LASSO + Cox
Wu et al., 2020 (50)	Eight-IRlncRNA immune-related signature (MIR181A2HG, AC114730.3, LINC00892, PTPRD-AS1, LINC01013, MRPL23-AS1, LINC01395 and AC002454.1)	High risk score → Worse OS	Tumor + scRNA	univariate Cox, LASSO
Studies that utilized computational validation cohort in addition to clinical Cohort				
Ma et al., 2021 (51)	Ti-lncRNA signature score: ENSG00000265148, ENSG00000281358, ENSG00000262089, ENSG00000240889, ENSG00000253230, ENSG00000261888, ENSG00000235304, ENSG00000226806 and ENSG00000260244	Low score → better PD-1 blockade response	Tumor/immune transcriptomes	Computational modeling and Cox
An et al., 2025 (52)	5-lncRNA ceRNA prognostic model: AP000695.2 AC010333.1 LINC01579 LINC00922 AL121772.1	Higher combined RiskScore → worse OS. Individual lncRNAs like AP000695.2 are higher expressed in more aggressive tumors. and reduced anti-PD-1 response	Tumor tissue	WGCNA + Cox + LASSO
Xue et al., 2022 (53)	19 candidates identified; one hub CDK1-related lncRNA highlighted (LINC00261)	low LINC00261 → immune resistance phenotype	Tumor transcriptomes	Bioinformatic correlation & infiltration analysis
Toker et al., 2023 (18)	NEAT1	High NEAT1 → enhanced IFNγ/inflammation → better ICI response in melanoma + longer survival in Glioblastoma	Tumor	NR
Ren et al., 2024 (54)	AC002331.1, CTA-384D8.35, RP11-291B21.2, AC006262.5, RP1-27K12.2 and RP11-54H7.4	Low risk → better OS/ICI response	Tumor transcriptomes	LASSO + Cox
Zhang et al., 2021 (55)	12 m6A-related lncRNA immune signature: (RNF217-AS1, RASAL2-AS1, PINK1-AS, SBF2-AS1, C14orf132, SNHG16, C17orf82, FAM13A-AS1, BDNF-AS, PSMB8-AS1, ZNRD1-AS1 and GUSBP11)	High → immunosuppressive TME & poor ICI response	Tumor RNA-seq	Pearson + LASSO
Song et al., 2022 (56)	Seven m6A- and m5C-related lncRNA prognostic signature: including 4 high-risk lncRNAs (NR2F1-AS1, ALMS1-IT1, NNT-AS1, and SNHG22) and 3 low-risk lncRNAs (LINC00628, STAM-AS1, and CASC2)	High-risk → worse prognosis Low risk → better OS; predicted better anti-PD-1/L1 response	Tumor transcriptomes	LASSO
Yang et al., 2024 (57)	10-lncRNA ferroptosis signature (AC099850.4, AL731567.1, AL133415.1, AC021321.1, SPAG5-AS1, HMGA2-AS1, RBMS3-AS3, AC006160.1, AL583785.1, and AL662844.4)	High risk → poor OS & immunosuppressive TME; Low risk → predicted ICI benefit	Tumor RNA-seq	LASSO + Cox
Jiang et al., 2021 (58)	Six stemness-related lncRNA signature (LINC01094, ADAMTS9-AS1, LINC01614, LINC00449, RNF144A-AS1, and MAPKAPK5-AS1)	High score → poor OS; Low score → higher likelihood of ICI benefit	Tumor RNA-seq	Cox + Lasso regression
Zhang et al., 2024 (59)	CYTOR	High CYTOR → poor OS & immune escape phenotype; differential in ICI responder clusters	Tumor + scRNA	qRT-PCR

ICI, Immune checkpoint inhibitor; OS, Overall Survival; WGCNA, Weighted Gene Co-expression Network Analysis; LASSO, Least Absolute Shrinkage and Selection Operator; Ti-LncRNA, tumor-infiltrating immune-related lncRNAs; GSEA, Gene Set Enrichment Analysis; m6A, N6-methyladenosine; m5C, 5-methylcytosine; ceRNA, competing endogenous RNA; RNA-seq, RNA sequencing; CDK-1, cyclin-dependent kinase 1; LUAD, Lung Adenocarcinoma; HNSCC, Head and Neck Squamous Cell Carcinoma; NMF, Non-negative Matrix Factorization; TME, Tumor Microenvironment; scRNA, Single-cell RNA; qRT-PCR, Real-time fluorescence quantitative PCR; HNSCC, Head and Neck Squamous Cell Carcinoma; SKCM, Skin cutaneous melanoma; m7G, N^7^-methylguanosine; PD-1, Programmed Death-1; PD-L1, Programmed Death-Ligand 1; CTLA-4, Cytotoxic T-Lymphocyte-Associated Protein-4, CYTOR, Cytoskeleton Regulator RNA; NR, not reported in the original publication despite review of the full text and **Supplementary Materials**.

### 3.4 lncRNA signature as prognostic biomarker

The majority of studies reported multi-lncRNA signatures, while only a few focused on individual lncRNAs (**Table 3**). In these models, patients classified as high-risk consistently showed poorer overall survival, whereas low-risk groups had more favorable outcomes. For studies evaluating single lncRNAs, increased expression was often associated with worse survival, although the direction of association varied depending on the transcript. Notably, several signatures derived from specific biological processes including ferroptosis- (35, 48), cuproptosis- (32, 47), m6A/m5C-related (37, 55, 56), and stemness-associated lncRNAs (58), demonstrated similar prognostic stratification patterns.

### 3.5 lncRNA signature as predictive biomarker

In addition to prognostic associations, many studies specifically evaluated the ability of lncRNA signatures to predict response to ICI (**Table 3**). Across studies, a highly consistent pattern was observed: Low-risk lncRNA scores were associated with improved response to ICI, including higher objective response rates and favorable disease control and high-risk scores were associated with reduced response or features consistent with resistance to immunotherapy. This predictive trend was observed across multiple datasets, including immunotherapy-treated cohorts such as IMvigor210, as well as across different cancer types and ICI regimens. However, one study reported that high-risk scores of m7G-related lncRNA signature were associated with increased immune checkpoint expression yet poorer survival, highlighting the complex role of lncRNAs in modulating immune activity and clinical outcomes (49). Interestingly, lncRNA expression showed association with tumor immune phenotypes. Signatures associated with better outcomes were typically found in immune-inflamed “hot” tumors with greater immune cell infiltration. In contrast, less favorable signatures were more often observed in immune-suppressive “cold” tumors with limited immune activity (39, 41, 47).

## 4 Discussion

This work reviewed the available evidence on the potential use of lncRNAs as predictive and prognostic biomarkers for ICI treatment. Across the included studies, lncRNA-based signatures showed prognostic value across multiple malignancies with high-risk scores generally associated with shorter OS and, where reported, poorer progression-free or disease-specific survival. Beyond their prognostic role, the predictive value of lncRNAs for ICI was also reported. Many of the included studies showed that lncRNA-based models were able to distinguish patients more likely to derive clinical benefit from ICI, with low-risk patients typically show better response rate. Low-risk lncRNA groups were commonly associated with better objective response, improved disease control, or more favorable predicted sensitivity to ICI treatment, whereas high-risk groups were more frequently linked to reduced response or features suggestive of therapeutic resistance. These findings highlight lncRNAs not only as markers of baseline prognosis but also as candidate tools for treatment stratification within the immunotherapy setting. Furthermore, lncRNA expression profiles were frequently linked to immune-related tumor characteristics. Favorable lncRNA signatures were often associated with immune-inflamed or “hot” tumors, whereas unfavorable signatures were more commonly linked to immune-suppressive or “cold” tumor states (39, 41, 47).

Although most included studies did not provide direct experimental mechanistic validation, some reported mechanistic or immune-related associations. For example, Toker et al. (18). demonstrated that elevated NEAT1 expression was associated with improved response to ICIs and increased IFNγ-related inflammatory signaling and immune-cell infiltration. These findings, together with existing mechanistic literature suggest that lncRNAs can function as important regulators of the immune landscape through modulation of the PD-1/PD-L1 axis, antigen presentation, inflammatory signaling, and immune-cell recruitment (16, 61–63). At the nucleus level, studies support its key role in chromatin, transcriptional regulation, mRNA stability, and RNA networks (64–66), all of which can shape tumor biology and immune cell signaling. Additionally, several of the studies included in this review linked specific lncRNAs to immune checkpoint regulation, CD8^+^ T-cell exhaustion, cytokine signaling, and tumor microenvironment (18, 39, 41, 42, 47, 49). These mechanisms provide a biologically rational for why lncRNA signatures constantly emerged as both prognostic and predictive markers in ICI-treated cancers.

Current established immunotherapy biomarkers such as PD-L1 expression, tumor mutational burden, and microsatellite instability have all been incorporated into clinical decision-making in selected settings, yet their performance remains limited, tumor-type dependent, and often insufficient to fully capture the complexity of tumor–immune interactions (67). By contrast, lncRNAs operate at the level of transcriptional and post-transcriptional regulation and may influence several immune-related pathways simultaneously. This broader regulatory capacity may explain why lncRNA-based signatures were repeatedly associated not only with survival and treatment response but also with tumor characteristics. Rather than functioning as isolated biomarkers, lncRNAs may therefore act as integrative indicators of tumor immunogenicity, immune suppression, or immune responsiveness.

The growing integration of ICIs into earlier treatment settings further highlights the need for reliable predictive biomarkers. For example, recent studies have demonstrated the feasibility and clinical activity of PD-1 inhibitor-based neoadjuvant strategies in locally advanced gastric cancer, reflecting the expanding role of immunotherapy across gastrointestinal malignancies. As the use of ICIs continues to broaden, the identification of biomarkers capable of predicting treatment response and patient outcomes will become increasingly important for optimizing patient selection and therapeutic benefit (68).

Several limitations should be acknowledged. First, most of the studies designed retrospectively, and many of which used publicly available datasets such as TCGA and GEO. This raises concerns regarding cohort overlap and selection bias. This overlap was particularly evident in TCGA-based studies within the same cancer type, including Head and Neck Squamous Cell Carcinoma (HNSCC) (39, 41, 51, 54), bladder cancer (35, 38, 43, 44, 49, 50, 55, 57), Lung Adenocarcinoma (LUAD) (33, 42, 53), gastric cancer (48, 52, 58), and melanoma (18, 38, 59). However, reported distinct lncRNA signatures and prognostic models despite substantial overlap in the underlying patient populations. This variability likely reflects differences in data preprocessing, feature-selection strategies, statistical modeling approaches, and biomarker selection criteria. Consequently, the reproducibility of individual lncRNA signatures remains uncertain. Furthermore, repeated development of lncRNA signatures within overlapping public datasets may increase the risk of overfitting, whereby models perform well in the datasets from which they were derived but may not generalize to broader patient populations. As a result, the external validity and clinical applicability of many proposed lncRNA biomarkers remain uncertain. Second, quantitative analysis was limited due to heterogeneity in lncRNA signature design. Third, many studies lacked real-world external cohort validation. Therefore, this limitation may restrict the utility of current findings in cancer patients. An additional consideration relates to the methodological quality assessment. The NOS was used because the majority of included studies were observational in nature and evaluated associations between lncRNA expression patterns or lncRNA-based risk signatures and clinical outcomes, including OS, PFS, and predicted ICI response. Although many studies utilized publicly available datasets such as TCGA, GEO, and IMvigor210, they retained the fundamental characteristics of retrospective cohort studies by comparing outcomes across patient groups defined by lncRNA expression levels or risk stratification models. Therefore, the NOS was considered an appropriate framework for evaluating study selection, comparability, and outcome assessment. Nevertheless, the NOS does not fully capture methodological challenges specific to bioinformatic investigations, including dataset reuse, batch effects, feature-selection bias, model overfitting, and limited external validation. Accordingly, the quality assessments reported in this review should be interpreted alongside these additional considerations when evaluating the strength and translational relevance of the evidence.

Another important limitation of the current literature is the frequent inability to distinguish prognostic from predictive biomarker effects. Although many studies reported significant associations between lncRNA signatures and survival outcomes in patients receiving ICIs relatively few incorporated non-immunotherapy comparator cohorts or formally evaluated treatment-biomarker interactions. Consequently, it is often difficult to determine whether these signatures specifically predict benefit from ICI therapy or simply reflect broader prognostic characteristics associated with patient outcomes. Future studies should prioritize prospective designs, inclusion of appropriate comparator populations, and formal interaction analyses to establish true treatment-specific predictive utility.

**Figure 4:**
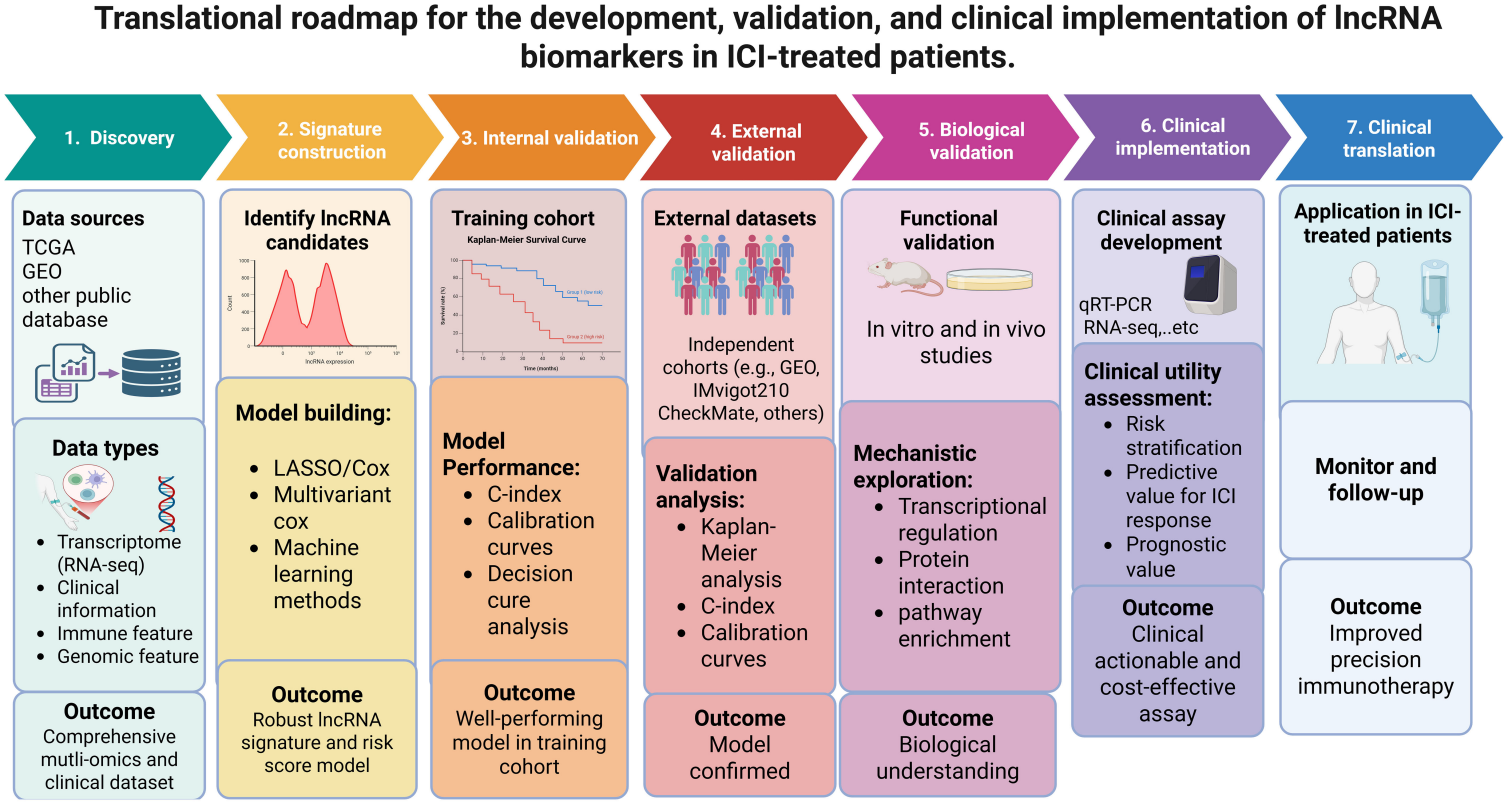
Translational Roadmap for the Development, Validation, and Clinical Implementation of lncRNA Biomarkers in ICI-Treated Patients. Proposed workflow for the development of clinically applicable lncRNA biomarkers, including biomarker discovery, signature construction, internal and external validation, biological validation, clinical assay development, and integration into clinical practice for patient stratification and prediction of immune checkpoint inhibitor response. lncRNA, long non-coding RNA; ICI, immune checkpoint inhibitor; TCGA, The Cancer Genome Atlas; GEO, Gene Expression Omnibus; RNA-seq, RNA sequencing; LASSO, least absolute shrinkage and selection operator; qRT-PCR, quantitative real-time polymerase chain reaction.

Importantly, the development of clinically applicable biomarkers requires substantially more evidence than the identification of statistically significant signatures in retrospective datasets. Although many of the included studies reported promising prognostic and predictive performance, most were derived from public transcriptomic datasets and lacked prospective validation. For a biomarker model to achieve translational relevance, several key steps are required, including validation in independent external cohorts, reproducible performance across different populations and sequencing platforms, prospective evaluation in patients receiving immune checkpoint inhibitors, and biological validation of the underlying mechanisms A proposed translational roadmap for the development, validation, and clinical implementation of lncRNA biomarkers in ICI-treated patients is presented in **Figure 4**. Recent advances in multi-omics integration and machine-learning approaches have further emphasized the importance of combining transcriptomic, genomic, and immune microenvironment data with robust validation frameworks to improve biomarker generalizability and clinical utility (69–71). Future lncRNA-based biomarkers should therefore move beyond retrospective computational modelling toward prospective and biologically validated frameworks capable of supporting clinical decision-making in immuno-oncology.

Future studies should focus on validation external cohort and consistent reporting of model development and determining cutoff threshold. In addition, further functional studies are needed to identify lncRNAs that play consistent roles across multiple cancer types. Overall, the findings of this systematic review support the growing recognition of lncRNAs as potential biomarkers in the era of precision immuno-oncology.

## Data Availability

The original contributions presented in the study are included in the article/**Supplementary Material**. Further inquiries can be directed to the corresponding author.
