# Supplementary figures and images for: Long non-coding RNAs and their potential role in predicting immunotherapy response and prognosis: a systematic review
Source: Front Immunol. 2026 Jul 10;17:1859747. doi: 10.3389/fimmu.2026.1859747 (PMC13396835; doi:10.3389/fimmu.2026.1859747)

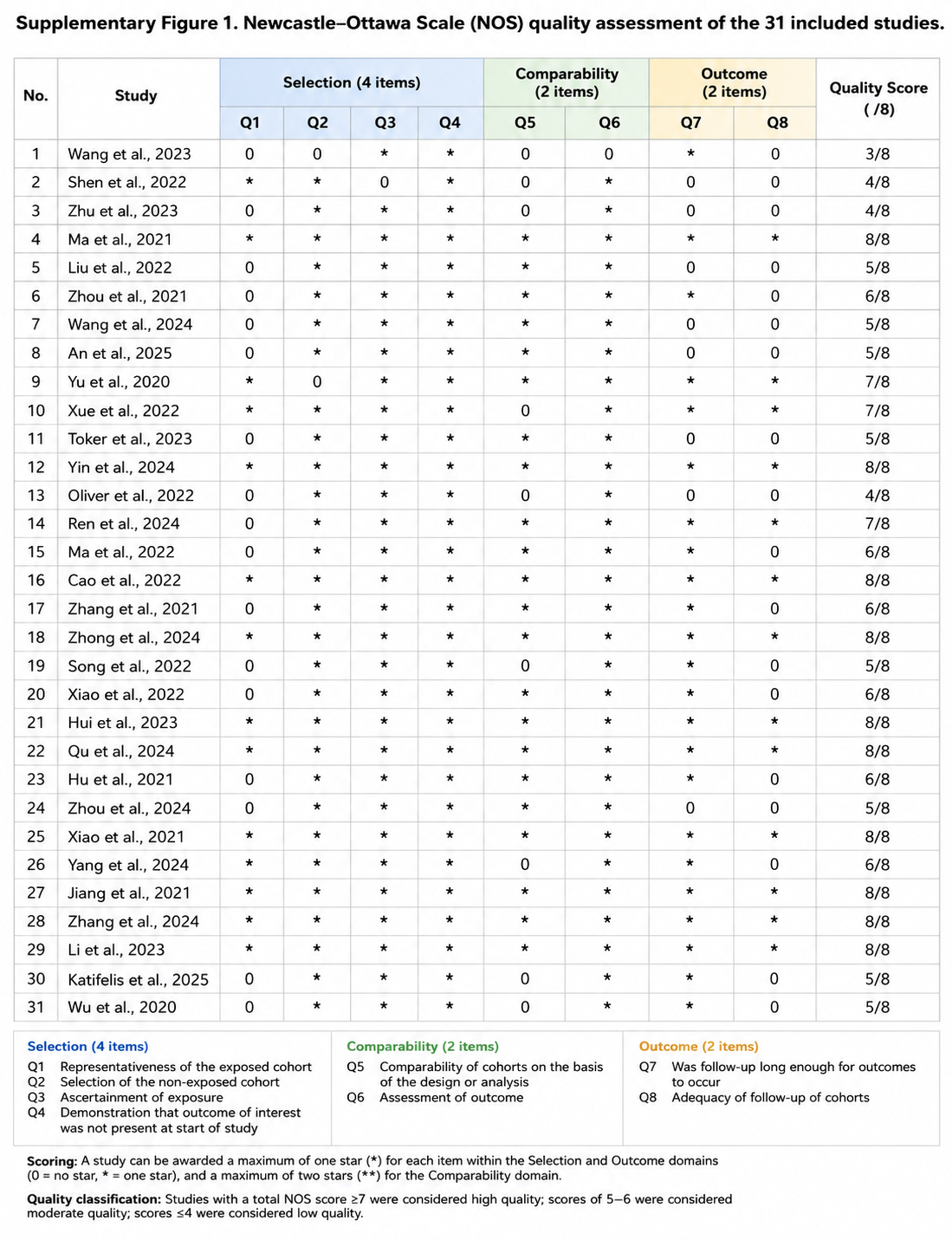

Supplement: Supplementary file 1 [file Image1.png]
