# Supplementary material for: Long non-coding RNAs and their potential role in predicting immunotherapy response and prognosis: a systematic review
Source: Front Immunol. 2026 Jul 10;17:1859747. doi: 10.3389/fimmu.2026.1859747 (PMC13396835; doi:10.3389/fimmu.2026.1859747)
Supplement: Supplementary file 2 [file Table1.docx]

Supplementary Tables and Figures

| **Supplementary Table S1. Detailed search strategies used in each database.** | |
| --- | --- |
| **Database** | **Search Strategy** |
| PubMed | ("long non-coding RNA"[Title/Abstract] OR lncRNA[Title/Abstract] OR lncRNAs[Title/Abstract] OR "long noncoding RNA"[Title/Abstract]) AND ("immune checkpoint inhibitor"[Title/Abstract] OR "immune checkpoint blockade"[Title/Abstract] OR immunotherapy[Title/Abstract] OR PD-1[Title/Abstract] OR PD-L1[Title/Abstract] OR CTLA-4[Title/Abstract] OR nivolumab[Title/Abstract] OR pembrolizumab[Title/Abstract] OR atezolizumab[Title/Abstract] OR durvalumab[Title/Abstract] OR avelumab[Title/Abstract] OR ipilimumab[Title/Abstract]) AND (cancer OR tumor OR tumour OR neoplasm OR malignancy) |
| MEDLINE | (("long non-coding RNA" OR "long noncoding RNA" OR lncRNA OR lncRNAs) AND ("immune checkpoint inhibitor" OR "immune checkpoint blockade" OR immunotherapy OR PD-1 OR PD-L1 OR CTLA-4 OR nivolumab OR pembrolizumab OR atezolizumab OR durvalumab OR avelumab OR ipilimumab) AND (cancer OR tumor OR tumour OR neoplasm OR malignancy)) |
| Web of Science | (("long non-coding RNA" OR "long noncoding RNA" OR lncRNA OR lncRNAs) AND ("immune checkpoint inhibitor" OR "immune checkpoint blockade" OR immunotherapy OR "PD-1" OR "PD-L1" OR "CTLA-4" OR nivolumab OR pembrolizumab OR atezolizumab OR durvalumab OR avelumab OR ipilimumab) AND (cancer OR tumor OR tumour OR neoplasm OR malignancy)) |
| **Abbreviations:** lncRNA, long non-coding RNA; PD-1, programmed cell death protein 1; PD-L1, programmed death-ligand 1; CTLA-4, cytotoxic T-lymphocyte-associated protein 4. | |
